# Dysregulated macrophage immunity in *Helicobacter pylori* infection: unveiling mechanistic insights and therapeutic implications

**DOI:** 10.3389/fimmu.2025.1636768

**Published:** 2025-08-04

**Authors:** Miao Xiang, Panpan Li, Xiaofei Yue, Linlin Liu, Linjing Wang, Nengjin Sun, Kaile Wang, Yuying Zhang, Hongyan Wang

**Affiliations:** ^1^ Key Laboratory of Immune Microenvironment and Inflammatory Disease Research in Universities of Shandong Province, School of Basic Medical Sciences, Shandong Second Medical University, Weifang, China; ^2^ Department of Pathogenic Biology, School of Basic Medical Sciences, Shandong Second Medical University, Weifang, China; ^3^ Rehabilitation Pharmacy Center, Beijing Rehabilitation Hospital, Capital Medical University, Beijing, China; ^4^ Department of Oncology, China-Japan Union Hospital, Jilin University, Changchun, China; ^5^ Department of Gastroenterology, Weifang People’s Hospital, Shandong Second Medical University, Weifang, China

**Keywords:** *Helicobacter pylori*, macrophages, phagocytosis, inflammation, antigen presentation, immunomodulation

## Abstract

*Helicobacter pylori* (*H. pylori*) is a microaerophilic, gram-negative spirochete that primarily colonizes the human gastric mucosa. It is strongly linked to gastritis, ulcers, and the development of malignant tumors. Macrophages, as one of the key components of the innate immune system, play a crucial role in maintaining immune homeostasis through a range of functions, including pathogen phagocytosis, antigen recognition and presentation, inflammation regulation and tumor immune surveillance. Emerging evidence suggests that *H. pylori* employs diverse molecular mechanisms to evade immune clearance by macrophages. This review provides a comprehensive analysis of how *H. pylori* infection modulates macrophage functions, including impairing pathogen recognition and phagocytosis, disrupting phagosome maturation and reducing immune clearance capacity. Furthermore, *H. pylori* infection skews macrophage polarization to promote chronic inflammatory damage, inhibits antigen processing and presentation to evade adaptive immune responses and induces macrophage apoptosis via activation of apoptotic signaling pathways. By unraveling the complex molecular interactions between *H. pylori* and macrophages, this review highlights strategies for reprogramming macrophage functions, offering innovative approaches to address the limitations of conventional antimicrobial therapies and advancing targeted therapeutic interventions for *H. pylori*-associated diseases.

## 1 Introduction

*Helicobacter pylori* (*H. pylori*) is a microaerobic, gram-negative spiral bacterium that colonizes the mucous membranes of the human gastrointestinal tract. This globally prevalent pathogen exhibited an infection rate of 58.2% between 1980 and 1990 (1). Although the prevalence has declined in recent years, it remains significantly high at 43.9% between 2015 and 2022 (2). Barry Marshall and Robin Warren first isolated and cultured this bacterium from the gastric mucous membranes of patients with peptic ulcers in 1983 (3). Research has demonstrated that *H. pylori* infection is a critical pathogenic factor in various gastrointestinal disorders, ranging from superficial gastritis and peptic ulcers to more severe conditions such as gastric adenocarcinoma and mucosa-associated lymphoid tissue (MALT) lymphoma (4). Consequently, *H. pylori* has been classified as a Group 1 carcinogen by the International Agency for Research on Cancer (IARC) of the World Health Organization (WHO), marking it as the first bacterium officially recognized as carcinogenic (5). The pathogenic mechanisms of *H. pylori* infection are exceedingly complex.

*H. pylori* infection exhibits pathogenic complexity attributable to its virulence factors, with studies confirming strong correlations between these factors and disease severity (6). Strains are classified as CagA-positive or -negative based on cytotoxin-associated gene A (CagA) expression; CagA-positive variants inject effector proteins into host cells via the type IV secretion system (T4SS), disrupting cytoskeletal dynamics and impairing cellular motility, proliferation, and apoptosis homeostasis. Conversely, ubiquitously expressed vacuolating toxin A (VacA) drives chronic colonization by inducing apoptosis, autophagy, membrane potential collapse, aberrant MAP kinase activation, and T-cell dysfunction (7–9). Urease, the most abundant *H. pylori* protein, serves dual functions: modulating pH to facilitate colonization while releasing ammonia to neutralize gastric acidity, and subverting host defenses through impaired opsonization, enhanced granulocyte chemotaxis, MHC-II-dependent apoptosis pathway activation, and pro-inflammatory cytokine storm amplification (10, 11). Lipopolysaccharide (LPS) evades pattern recognition receptors via lipid A/O-antigen structures, perpetually activating NF-κB-mediated inflammatory cascades that accelerate the gastritis-ulcer-carcinogenesis sequence (12, 13). The flagellum, essential for motility and chemotaxis, mediates initial mucosal colonization while modifying TLR5 signaling to promote inflammatory polarization and immune evasion (14, 15). Collectively, these virulence factors enable gastric mucosal persistence, subvert immune responses, drive immune escape, and ultimately induce carcinogenesis (16).

Microglia arise directly from yolk sac progenitors during early embryogenesis, whereas most other tissue-resident macrophages are derived from fetal liver monocytes generated through embryonic hematopoiesis (17). In adults, circulating macrophages typically originate from bone marrow–derived monocytes, particularly under inflammatory conditions (17). As a crucial component of the innate immune system, macrophages exhibit potent phagocytic abilities. While both macrophages and neutrophils share phagocytic functions, macrophages have a longer lifespan. In response to pathogen invasion, macrophages act as “scavengers” and, along with neutrophils, are the first responders to infection. As a crucial component of innate immunity, their phagocytic abilities enable direct engulfment and destruction of invading microbes. The pattern recognition receptors (PRRs) on the surface of macrophages recognize pathogen-associated molecular patterns (PAMPs), triggering innate immune signaling pathways to exert antimicrobial effects (18). Beyond their role in pathogen clearance, macrophages also promote inflammation process, present antigens and contribute to tumor immunity, thereby maintaining immune homeostasis (19, 20). Although macrophages serve as the first line of defense against invading pathogens, certain microbes, such as *H. pylori*, have evolved mechanisms to disrupt the immune regulatory functions of macrophages, thereby facilitating the persistence of infection and contributing to tumorigenesis (21–24). This review explores the mechanisms by which *H. pylori* infection reprograms macrophage functionality, providing innovative solutions to overcome the limitations of conventional antimicrobial therapy, which may inform macrophage-centric therapeutic development for *H. pylori*-associated disorders.

## 2 *H. pylori* infection and macrophage immune dysfunction: mechanisms and implications

### 2.1 The impact of *H. pylori* infection on macrophage recognition and phagocytosis of pathogens

**Figure 1:**
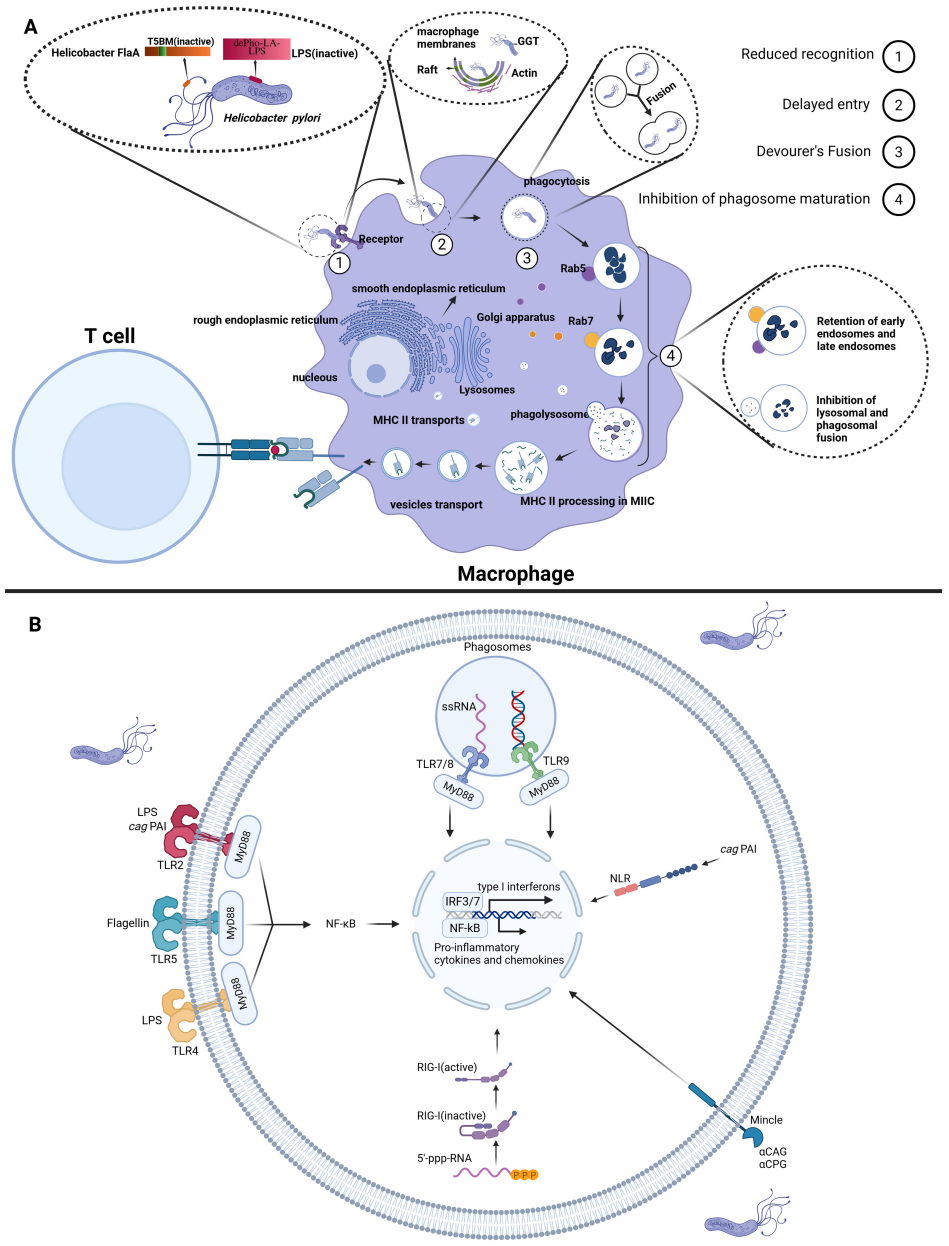
The impact of *H. pylori* on macrophage recognition and phagocytic function. **(A)** (1) Reduced Recognition: *H. pylori* diminishes macrophage PRR-mediated recognition by expressing O-antigens that closely resemble human antigens and through mutations in flagellin (FlaA). (2) Delayed Entry: *H. pylori* synthesizes CGs, which activates the PI3K pathway, regulating actin dynamics and thereby delaying macrophage phagocytosis. (3) Fusion of Phagocytes: *H. pylori* promotes the fusion of macrophages that have internalized the bacteria, thereby disrupting normal phagosome maturation and delaying the clearance of the pathogen. (4) Inhibition of Phagosome Maturation: *H. pylori* enhances resistance in early and late endosomes and lysosomes via GGT; Additionally, it recruits Coronin 1A to delay the formation of phagolysosomes. **(B)** Recognition of *H. pylori* by innate immune cells. Created with Biorender.com.

Upon *H. pylori* infection, innate immune cells such as macrophages recognize bacterial PAMPs through PRRs, including Toll-like receptors. Following recognition, macrophages extend their plasma membranes to engulf *H. pylori*, forming membrane-bound phagosomes (25). Subsequently, the phagosomes sequentially interact with early and late endosomes, followed by fusion with lysosomes to form phagolysosomes. These phagolysosomes contain fully functional lysosomal hydrolases, potent enzymes capable of degrading pathogens (26, 27). During digestion by hydrolases, small peptides are generated that form complexes with MHC-II. These peptide-MHC-II complexes are then transported via vesicles to the cell surface, where they are presented for antigen recognition (28) (**Figure 1A**).

The initial response of macrophages to *H. pylori* infection is characterized by the specific recognition of PAMPs by PRRs, including Toll-like receptors (TLRs), intracellular RIG-I-like receptors (RLRs), C-type lectin receptors (CLRs) and nucleotide oligomerization domain (NOD)-like receptors (NLRs). TLR4, TLR2 and TLR5 on innate immune cell membranes recognize *H. pylori*-derived LPS and flagellin, respectively. This recognition triggers activation of the NF-κB signaling pathway and inflammasome complexes, orchestrating the expression of pro-inflammatory cytokines and chemokines (29, 30). The intracellular recognition mechanisms demonstrate greater complexity: TLR9 recognizes unmethylated CpG DNA, while TLR7/8 specifically identifies bacterial single-stranded RNA, with both converging on the MyD88-TRIF signaling axis to activate immune responses (31–33). Concurrently, RIG-I senses 5’-triphosphorylated RNA (5’-ppp-RNA) to initiate type I interferon production (18, 32). Emerging research reveals that the cytotoxin-associated gene pathogenicity island (*cag* PAI) of *H. pylori* precisely orchestrates IL-1β synthesis and secretion through synergistic interactions with TLR2, NOD2 and NLRP3 (34). CLRs recognize Cholesteryl acyl α-glucoside (αCAG) and cholesteryl phosphatidyl α-glucoside (αCPG) of *H. pylori* to exacerbate inflammation (35) (**Figure 1B**). However, certain PAMPs of *H. pylori* can evade recognition by macrophage PRRs due to their weak immunogenicity. For instance, *H. pylori* evades detection by PRRs through the expression of O-antigens that resemble human antigens and the production of low-activity LPS endotoxins (36). Additionally, flagellin, which normally serves as the ligand for TLR5, is recognized as a pro-inflammatory trigger upon detection of the flagella of *H. pylori*. However, mutations in *H. pylori* flagellin (FlaA) prevent TLR5 from recognizing the bacterium’s flagellin (37).

Upon successful recognition of *H. pylori* by macrophages, the cholesterol-α-glucosyltransferase (CGT) of type I *H. pylori* synthesizes and inserts cholesteryl glucosides (CGs) into the lipid raft regions at the *H. pylori*-macrophage attachment sites. This action activates class I phosphoinositide 3-kinases (PI3Ks), which regulate actin polymerization and thus delay phagocytosis by macrophages (38–40).

Following the recognition and internalization of *H. pylori*, the phagosome promptly enters the maturation phase. Experimental evidence demonstrates that phagosomes containing individual *H. pylori* may undergo homotypic fusion, leading to the formation of megasomes. These megasomes disrupt the normal metabolic processes of both early and late endosomes, thereby impeding maturation of phagosomes (39, 41, 42). Nevertheless, the precise molecular mechanisms underlying the formation of megasomes remain incompletely understood. Early studies suggested that the VacA and/or *cag* PAI might contribute to this process. However, subsequent research has demonstrated that megasome formation is independent of both VacA and the *cag* PAI of *H. pylori*. Instead, the presence of urease has been found to impair the aggregation and fusion of phagosomes (39, 43, 44). In addition to promoting megasome formation, *H. pylori* GGT has been shown to enhance the resistance of both early and late endosomes, as well as lysosomes, thereby delaying phagolysosome formation (38, 45). Furthermore, *H. pylori* can directly disrupt the fusion of phagosomes with lysosomes in macrophages by recruiting and retaining Coronin 1A, thus impeding phagosome maturation (46).

These mechanisms demonstrate that *H. pylori* evades macrophage clearance by comprehensively inhibiting recognition, phagocytosis, digestion and degradation (**Figure 1A**). Despite substantial research efforts to elucidate *H. pylori*’s strategies for evading macrophage recognition and phagocytosis, the intricate nature of its PAMPs remains a key research focus.

### 2.2 The pathogenic role of *H. pylori* infection on macrophage-mediated inflammatory regulation

Macrophages are inherently heterogeneous and plastic, making them crucial regulators of inflammatory processes and significant contributors to both the initiation and resolution of inflammation (47). Upon exposure to diverse stimuli, macrophages undergo polarization into distinct activation subtypes, each exhibiting unique phenotypic characteristics and cytokine secretion profiles. Classically activated M1 macrophages primarily secrete inflammatory mediators to execute immune defense and combat pathogen invasion. In contrast, alternatively, activated M2 macrophages produce anti-inflammatory factors, promoting tissue repair and wound healing. Recently, beyond the well-characterized M1 and M2 subtypes, regulatory macrophages (Mreg) have been identified. These cells are activated by LPS in combination with immune complexes and secrete anti-inflammatory factors, playing a role in immune regulation (19, 48, 49).

**Figure 2:**
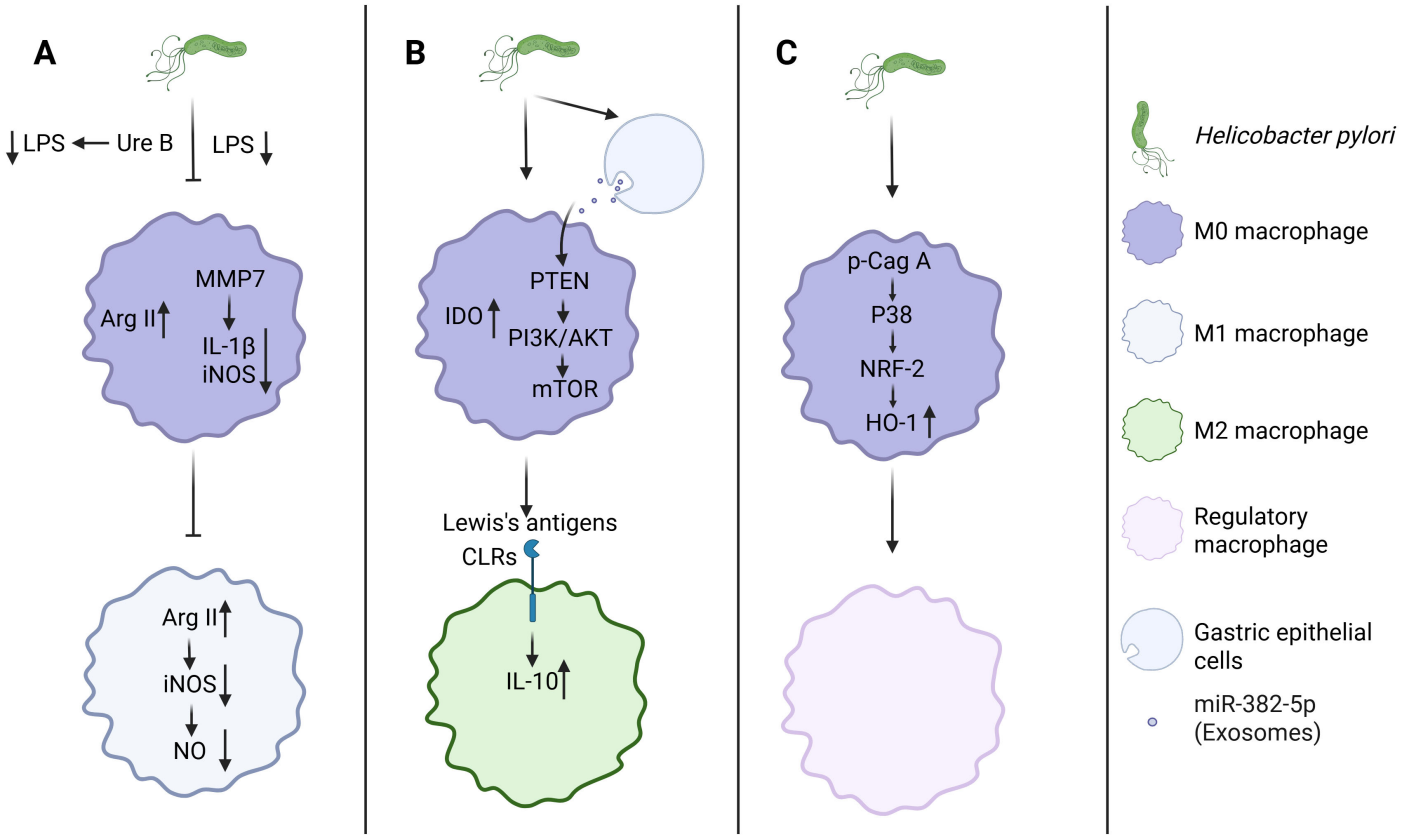
*H. pylori* orchestrates macrophage polarization through triple-pathway regulation. **(A)** *H. pylori* inhibits M1 macrophage polarization. **(B, C)** *H. pylori* promotes M2, Mreg macrophage polarization. Created with Biorender.com.

During pathogen infection, macrophages in the gastric mucosa are recruited and activated into M1 macrophages, which produce numerous inflammatory mediators to execute immune defense (48). For example, during *Mycobacterium tuberculosis* (Mtb) infection, M1 macrophages upregulate innate immune regulatory genes to facilitate the degradation of Mtb (50). However, the Mtb effector PPE36 can inhibit the activation of the ERK signaling pathway, thereby suppressing M1 macrophage polarization and reducing the production of pro-inflammatory cytokines (51). Similarly, *Salmonella typhimurium* utilizes the type III secretion system (T3SS) encoded by pathogenicity island 1 (SPI-1) and SPI-2 to enhance its survival and replication within macrophages (52). Studies have demonstrated that *H. pylori* can also inhibit or attenuate the activation of M1-type macrophages through multiple mechanisms, thereby achieving immune evasion and reducing inflammatory responses (**Figure 2**). The activity of *H. pylori*’s LPS is significantly lower compared to other Gram-negative bacteria such as *Escherichia coli*, leading to a diminished potential for M1 macrophage activation (36). Moreover, *H. pylori* can also inhibit M1-type macrophage polarization through enzymatic activity. Specifically, the bacterium’s urease subunit B (UreB) attenuates LPS-induced M1 polarization in macrophages (53). Through the action of matrix metalloproteinase 7 (MMP7), *H. pylori* downregulates IL-1β and inducible nitric oxide synthase (iNOS) mRNA expression in macrophages, thereby further impeding M1 polarization (53, 54). Arginase(Arg) plays a crucial role in the host’s anti-inflammatory response (55). *H. pylori* upregulates Arg2 expression in macrophages, which subsequently restricts M1 macrophage activation (56). This upregulation of Arg2 additionally reduces iNOS protein levels, leading to decreased nitric oxide production and consequently impairing macrophage-mediated bactericidal activity against *H. pylori* (57).

Unlike their pro-inflammatory M1 counterparts, M2 and Mreg actively produce anti-inflammatory mediators that help to suppress inflammatory pathways (58). Stimulating the polarization of macrophages towards the M2 or Mreg phenotype appears to be another strategy employed by *H. pylori* to evade immune clearance by macrophages (59). Research led by Alain P. Gobert et al. demonstrated that *H. pylori* can trigger the expression of heme oxygenase 1 (HO-1) in murine macrophages via the p-CagA/p38/NRF-2 signaling axis, thereby promoting the polarization of macrophages towards the Mreg phenotype (60). Additionally, Devi et al. demonstrated that *H. pylori* LPS Lewis antigens engage CLRs to induce IL-10 production in macrophages (61). Peng et al. indicated that *H. pylori* upregulates the expression of indoleamine 2,3-dioxygenase (IDO) in macrophages, further driving their polarization towards the M2 phenotype (62). Notably, *H. pylori*-infected gastric cancer cells secrete exosomal miR-382-5p that targets the PTEN gene in macrophages, thereby activating the PI3K/AKT/mTOR signaling pathway to inhibit autophagy and promote macrophage M2 polarization (63). Despite evidence that *H. pylori* evades immune surveillance by polarizing macrophages towards M2 or Mreg phenotypes, current research in this area is still in its early stages, with the molecular mechanisms and regulatory networks that need to be fully elucidated.

### 2.3 The influence of *H. pylori* infection on antigen processing and presentation

As crucial components of the innate immune system, macrophages function as antigen-presenting cells (APCs) akin to dendritic cells, facilitating the presentation of antigens to T lymphocytes (20, 64). Macrophages internalize antigens through mechanisms such as phagocytosis, trogocytosis, endocytosis and pinocytosis, and subsequently present these antigens to T cells via cross-presentation (65). This antigen-presenting capacity is crucial for macrophages to regulate immune responses. However, *H. pylori* infection disrupts the antigen recognition and phagocytic functions of macrophages, thereby affecting antigen uptake. Furthermore, *H. pylori* impairs the antigen-presenting capacity of macrophages by modulating intracellular miRNA expression. Studies have elucidated that *H. pylori* downregulates miR-4270 in macrophages, which in turn upregulates the immune receptor CD300E, leading to decreased surface expression of MHC-II molecules (66). This hampers the recognition and activation of effector T cells, thereby diminishing the antigen-presenting efficacy of macrophages (66). The application of miRNA microarray profiling in macrophages has identified that MHC-II downregulation post-*H. pylori* infection is chiefly mediated by the suppression of class II major histocompatibility complex transactivator (CIITA) expression (67). CIITA, a critical regulator of MHC-II gene transcription, is modulated by multiple miRNAs. Studies have demonstrated that *H. pylori* upregulates miRNAs such as let-7i-5p, miR-146b-5p and miR-185-5p, which inhibit CIITA expression, thereby suppressing the synthesis of HLA-II molecules and further decreasing the antigen-presenting capacity of macrophages (67). Additional studies have demonstrated that *H. pylori* utilizes ADP-heptose, an intermediate metabolite in the LPS biosynthesis pathway, to upregulate miR-146b expression (68). This leads to CIITA downregulation and subsequent suppression of HLA-II expression, resulting in impaired antigen-presenting function (68). This elucidates *H. pylori*’s immune evasion strategy through the precise modulation of miRNA expression to impair macrophage antigen-presenting capabilities. Moreover, recent findings indicate that UreB can also inhibit macrophage antigen presentation via its interaction with TLR2 (53). Further studies indicate that *H. pylori* can also modulate macrophage antigen presentation and immune responses by altering their differentiation states and modulating activation patterns. The differentiation status of macrophages significantly influences their immunological functions, consequently affecting both the efficiency of antigen presentation and the potential for T cell activation (69).

**Figure 3:**
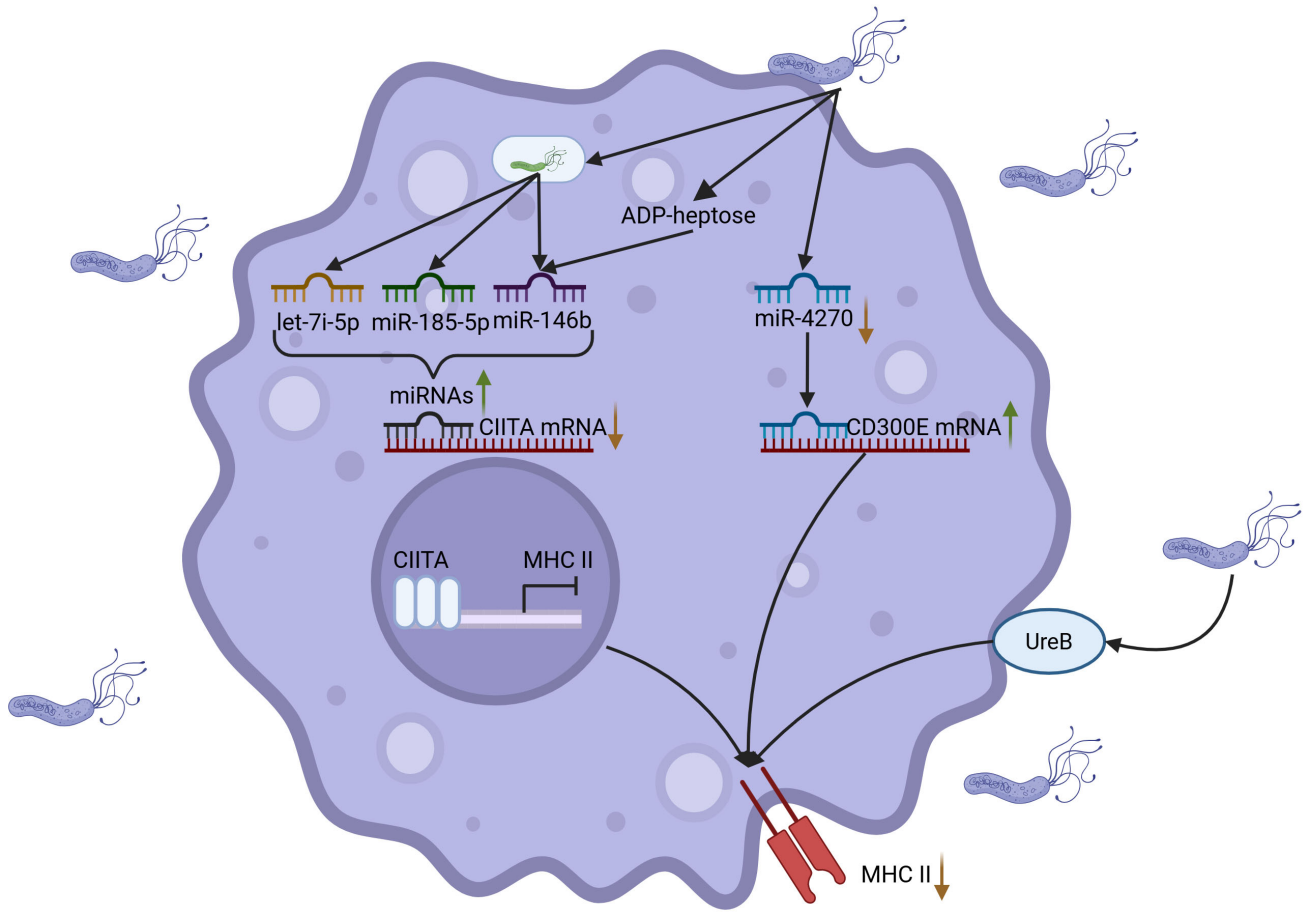
*H. pylori* inhibits antigen presentation of macrophages. *H. pylori* suppresses macrophage antigen presentation through coordinated mechanisms: (1) *H. pylori* downregulates miR-4270 to induce upregulation of CD300E, leading to diminished MHC-II surface expression; (2) *H. pylori* upregulates miRNAs (including let-7f-5p and miR-146b) to inhibit the transcriptional regulator CIITA, thereby impeding MHC-II biosynthesis; (3) Synergistic action of the metabolite ADP-heptose and surface protein UreB-TLR2 receptor interaction cooperatively impairs antigen-presenting capacity, ultimately facilitating immune evasion. Created with Biorender.com.

Consequently, *H. pylori* utilizes a complex immune evasion strategy by modulating macrophage miRNA expression, MHC-II molecule presentation, macrophage subtype differentiation and the action of virulence factors (**Figure 3**). Nevertheless, a comprehensive elucidation of the mechanisms by which *H. pylori* infection impacts macrophage antigen-presenting function remains to be fully achieved.

### 2.4 The impact of *H. pylori* infection on macrophage apoptosis

**Figure 4:**
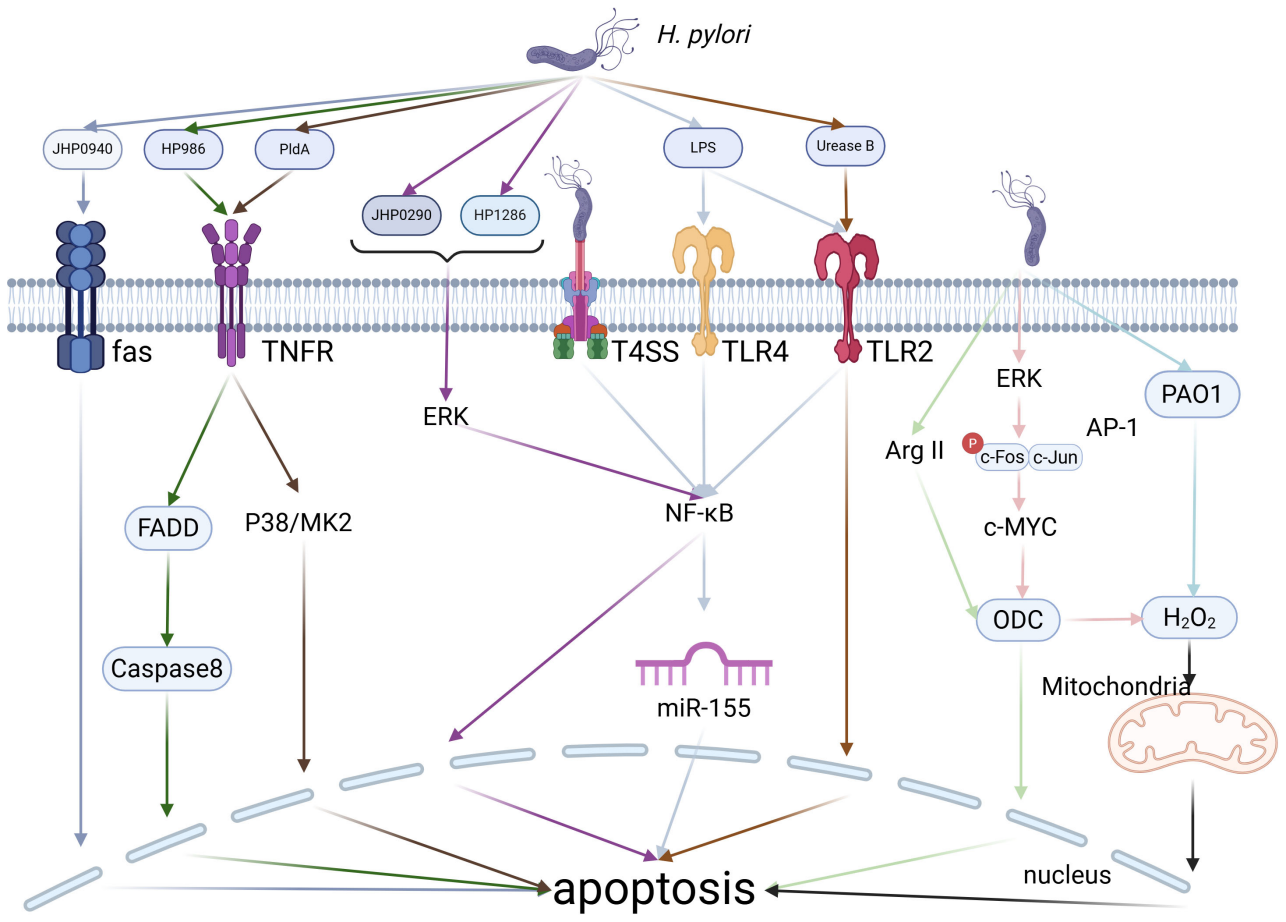
*H. pylori*-Induced Apoptosis. *H. pylori* can induce macrophage apoptosis through various pathways, including its own secreted proteins, enzymes, or the modulation of intracellular enzymes and microRNAs within macrophages. Created with biorender.com.

*H. pylori* infection modulates the immune microenvironment of gastric cancers through multiple pathways, particularly via induction of macrophage apoptosis, which significantly impairs macrophage function (**Figure 4**). As essential cells in immune surveillance, macrophages play a pivotal role in anti-tumor immunity. Therefore, any disruption in their apoptotic processes can profoundly affect tumor initiation and progression. Studies have demonstrated that *H. pylori* can trigger macrophage apoptosis through diverse molecular pathways. Initially, *H. pylori* triggers macrophage apoptosis by activating Arg2 and c-Myc-induced ornithine decarboxylase (ODC) (70, 71). Subsequent research by Menaker RJ et al. demonstrated that *H. pylori*-induced macrophage apoptosis is associated with alterations in the mitochondrial pathway (72). Furthermore, studies by Chaturvedi R and Asim M et al. revealed that the activation of polyamine oxidase 1 (PAO1) by *H. pylori* leads to the production of H_2_O_2_, which induces macrophage apoptosis through a mitochondrial-dependent mechanism. Simultaneously, *H. pylori* activates ERK1/2, leading to the formation of an AP-1 complex that binds to c-Myc, thereby upregulating ODC transcription and generating H_2_O_2_, which subsequently triggers apoptosis via mitochondrial membrane depolarization (73, 74). Additionally, *H. pylori* produces several secretory proteins that regulate macrophage apoptosis. For instance, JHP940 and HP986 from *H. pylori* trigger apoptosis through Fas and TNFR pathways, while JHP0290 and HP1286 promote c-Myc transcription by forming the AP-1 transcription factor complex via the ERK/MAPK signaling pathway, leading to macrophage apoptosis (75–78). As research advances, more mechanisms involved in *H. pylori*-induced macrophage apoptosis are being discovered. For example, *H. pylori* secretes phospholipase A (PldA), which triggers macrophage apoptosis via the p38-MK2 signaling pathway. Additionally, UreB can facilitate macrophage apoptosis by binding to TLR2, and VacA induces macrophage apoptosis through NF-κB activation (53, 79, 80). In addition, *H. pylori* infection in macrophages induces apoptosis through LPS interactions with TLR2 and TLR4, along with the upregulation of miR-155 via the T4SS. miR-155 promotes macrophage apoptosis by regulating the expression of several apoptosis-related genes, including Tspan14, SOCS1 and TNF-α (81, 82). In conclusion, *H. pylori* triggers macrophage apoptosis via various mechanisms, which impairs their role as anti-tumor immune surveillance cells, thus promoting the initiation and progression of gastric cancers. This intricate impact is orchestrated via the activation of intracellular signaling pathways and epigenetic modifications, particularly through microRNA regulation, highlighting the bacterium’s crucial role in tumor immune evasion strategies.

## 3 The role of macrophages in gastric cancer immunity

*H. pylori* infection is a major independent risk factor for gastric cancer (GC), with approximately 1-2% of those infected progressing to GC. The development of GC is a complex, multi-step process influenced by various factors, with the tumor microenvironment (TME) playing a crucial role (83). The TME is composed of evolving tumor cells and a complex stromal matrix. This stromal component includes diverse cellular constituents, notably tumor-associated macrophages (TAMs), which represent a significant component of the tumor stroma (84, 85). Circulating monocytes are recruited to the tumor periphery through the action of various chemokines and cytokines secreted by tumor cells or stromal cells. Upon infiltrating tumor tissues or clustering within the solid tumor microenvironment, these macrophages are defined as TAMs (86).

TAMs constitute the principal population of innate immune cells within the tumor immune microenvironment, exhibiting functional versatility with both tumor-promoting and tumor-suppressive potentials mediated via distinct immunological pathways (87–89). *H. pylori* infection is recognized as a major risk factor for GC development, primarily influencing TAMs by altering macrophage polarization states. Studies have shown that *H. pylori* infection can disrupt the differentiation of M1 macrophages and promote the formation of M2 macrophages, or even trigger the transdifferentiation of M1 macrophages into M2 macrophages. Subsequently, M2 macrophages further contribute to tumorigenesis and progression by promoting angiogenesis and secreting immunosuppressive factors (90, 91). Research by Bingting Yu et al. demonstrated that *H. pylori* infection triggers an inflammatory phenotype associated with M1 polarization. However, during chronic infection, sustained autocrine/paracrine IL-6 stimulation promotes the polarization of macrophages towards an M2 phenotype, potentially contributing to the development of gastric cancer (92). Recent studies have also revealed that chronic inflammation induced by M1-like TAM activity may accelerate genomic instability in malignant cells under certain conditions, serving as a driver of tumor progression (93). Furthermore, following *H. pylori* infection, gastric cancer cells secrete exosomes that subsequently transfer to adjacent immune cells, thereby altering macrophage function (63). This interaction between gastric cancer cells and immune cells highlights the complex interplay between the immune system and tumorigenesis in the context of chronic infection.

Macrophages exposed to these exosomes tend to preferentially secrete the pro-inflammatory cytokine IL-1β and promote tumor cell proliferation, migration and invasion by activating the Akt and MAPK signaling pathways. Since IL-1β plays a critical role in driving the proliferation, migration and invasion of malignant tumors, it is important to further investigate the mechanisms underlying this process (94).

While the tumor-promoting role of TAMs in gastric cancer progression, particularly in relation to *H. pylori* infection, has garnered considerable attention, further exploration is needed to understand their dual functions within the tumor immune microenvironment. Most studies currently focus on how TAMs promote tumor growth through immunosuppressive mechanisms in the tumor microenvironment. However, TAMs may also exhibit tumor-suppressive potential under certain conditions. Notably, during *H. pylori* infection, TAMs can be reactivated, leading to the phagocytosis and cytotoxic eradication of malignant cells.

## 4 Therapeutic approaches for *H. pylori* infection and macrophage-directed treatment

Currently, common regimens for treating *H. pylori* infection include a combination of bismuth agents, proton pump inhibitors (PPIs) and antibiotics like metronidazole and clarithromycin (95, 96). Proton pump inhibitors (PPIs), considered the cornerstone drugs in conventional therapy, are widely used in *H. pylori* treatment for their ability to inhibit *H. pylori* growth, increase gastric pH, enhance antibiotic concentration in gastric tissues and improve antimicrobial efficacy (97). However, due to the extensive use of antibiotics over the years, the resistance rates of *H. pylori* to various antibiotics have significantly increased (98). In particular, the rising resistance to clarithromycin, metronidazole and levofloxacin has led to a gradual decline in the efficacy of traditional triple or quadruple therapies (99). The growing problem of antimicrobial resistance makes the development of new treatment strategies essential (100). Macrophages, crucial components of the innate immune system, display significant flexibility and are essential in fighting pathogen infections and aiding tissue repair. Notably, macrophages play a crucial role in the immune clearance of *H. pylori*, as well as in the development of chronic inflammation, gastric mucosal damage and the progression to gastric cancer caused by *H. pylori* infection (101, 102). Therefore, macrophages have become a novel therapeutic target for *H. pylori* treatment (see **Table 1**). Certain pharmacological agents and natural compounds have been identified to modulate macrophage function, thereby enhancing the therapeutic efficacy against *H. pylori* infection. Specifically, agents that augment the pathogen-clearing ability of macrophages, such as patchouli alcohol, can significantly reduce the *H. pylori* ‘s acid resistance, antibiotic resistance and gastric colonization capacity (103). Furthermore, as research progresses, new therapeutic strategies are gradually emerging. For instance, studies reveal that silymarin, naphthopyranone compounds, and specific probiotics (*Lactobacillus gasseri* Kx110A1, *Lacticaseibacillus rhamnosus* UCO-25A and *Lactobacillus fermentum* UCO-979C) effectively suppress macrophage release of pro-inflammatory cytokines such as TNF-α, IL-6, and IL-8. This inhibition consequently prevents inflammatory tissue damage and promotes gastric mucosal repair (104–108). Notably, the probiotic *Lacticaseibacillus rhamnosus* UCO-25A further modulates the balance between pro-inflammatory and anti-inflammatory factors and promote the production of IL-10, thereby enhancing immunomodulatory effects. Additionally, certain natural compounds like berberine, quercetin and *Weizmannia coagulans* BCF-01 can inhibit the polarization of macrophages toward the M1 phenotype, thus suppressing inflammation-induced damage (109–111). Moreover, recent studies have found that lactate, a metabolic byproduct, exerts immunomodulatory effects by inhibiting the release of inflammatory factors from macrophages induced by *H. pylori*. However, its potential as a therapeutic option remains to be further investigated (112).

**Table 1:** Anti-*H. pylori* drugs targeting macrophage.

Categories	Function	Reference
Compounds		
patchouli alcohol	The intervention enhances the digestion and clearance of *H. pylori* by macrophages without affecting their phagocytic activity.	(103)
Berberine	During *H. pylori* infection, it modulates M2 macrophage polarization through the IL-4-STAT6 signaling pathway, contributing to the clearance of *H. pylori* and the treatment of chronic atrophic gastritis.	(109)
Silibinin	It suppresses the production and release of TNF-α and IL-6 in macrophages induced by *H. pylori*, thereby controlling infection-related inflammatory responses and preventing pathological changes.	(104)
Naphthopyranones	It inhibits the production of pro-inflammatory cytokines and nitric oxide in macrophages, thereby modulating inflammatory responses.	(105)
Quercetin	It mitigates inflammatory damage caused by macrophage M1 polarization through the SP1/LCN2 axis.	(110)
Probiotics		
*Lactobacillus gasseri* Kx110A1	*Lactobacillus gasseri Kx110A1* directly acts on macrophages by downregulating the expression of ADAM17 (also known as TNF-α-converting enzyme, TACE), thereby inhibiting the release of TNF and IL-6 and exerting anti-inflammatory effects.	(106)
*Weizmannia coagulans* BCF-01	It inhibits the differentiation of pro-inflammatory macrophages and pro-inflammatory CD4+ T cells.	(111)
*Lacticaseibacillus rhamnosus* UCO-25A	By forming a biofilm on the cell surface, it reduces the production of inflammatory factors such as TNF-α and IL-8 by gastric epithelial cells and macrophages, exhibiting anti-inflammatory effects. Additionally, it promotes the production of the immunoregulatory cytokine IL-10 by macrophages, contributing to the appropriate balance between inflammatory and anti-inflammatory factors. This mechanism aids in pathogen clearance, prevents chronic infection and mitigates inflammatory tissue damage, demonstrating beneficial therapeutic potential.	(107)
*Lactobacillus fermentum* UCO-979C	It induces a distinct balance of TNF-α, IFN-γ and IL-10 production in macrophages, providing protective effects against *H. pylori* infection by enhancing pathogen clearance and safeguarding against inflammatory damage.	(108)

Although targeting macrophages for the treatment of *H. pylori* infection has made some progress, numerous challenges still exist. In drug research, current probiotics are derived from various origins, with limited studies focusing on strains isolated from human gastric mucosa. Utilizing strains from other sources carries significant risks. The long-term application of probiotic interventions may pose a risk of gut microbiota imbalance (113). Moreover, while probiotics act on macrophages to clear *H. pylori*, *H. pylori* also produces virulence factors to inhibit the function of probiotics. Furthermore, the specific substances within probiotics that contribute to their anti-*H. pylor*i effects have yet to be fully understood. Additionally, most current compounds are limited to *in vitro* experiments or small-scale *in vivo* studies, highlighting a significant gap before practical applications can be achieved. In research on macrophages, their high plasticity offers opportunities for targeted treatments while also presenting significant challenges. The mechanisms underlying the differentiation of macrophages during *H. pylori* infection remain incompletely elucidated, potentially resulting in instability during therapeutic interventions.

## 5 Conclusion

In summary, the influence of *H. pylori* infection on the immunoregulatory functions of macrophages is highly complex. Macrophages, as key components of the immune system, play a crucial role in maintaining immune homeostasis and influencing disease susceptibility. *H. pylori* establishes persistent infection through sophisticated modulation of macrophage functions, including pattern recognition, phagocytic activity, inflammatory regulation, antigen presentation and tumorigenic processes.

Moreover, treating *H. pylori* infection continues to face multiple challenges, especially due to increasing antibiotic resistance. Hence, modulating macrophage functions, especially by inhibiting excessive inflammatory responses or promoting their reparative capabilities, may offer novel treatment directions. While significant insights have been obtained, many aspects of the interaction between *H. pylori* and macrophages remain unclear, highlighting the need for further research to develop new immunotherapy approaches against *H. pylori*.
